# Dissection Wounds

**Published:** 1860-04

**Authors:** W. C. Nichols


### *Dissection Wounds.* By W. C. Nichols, M. D.

The diligent student of anatomy is often troubled with the reflection that the acquisition of knowledge in this department of science is replete with difficulties. Perhaps by stealth, and at midnight, he procures the material suited to his purpose; and were he not led on by elevated aims, he might abhor this encroachment on the sanctity of the tomb. Thus it happens that many in passing along the intricate paths of this science, consider the way too toilsome, and deem the value of the acquisition hardly commensurate with the hazard of the adventure.—Though filled with repugnance at the mutilated body, the student must reflect that all learning is slow in coming to maturity. Attempts must be made barren of success, experiments without satisfactory results; and it must be early discovered, that, as there is a vast difference between the outlines of a shadow and the life-like picture of the master, so there is a wonderful distinction between the first rudiments of an art and its extreme perfection—between cultivated fields and fallow ground.

Being constantly engaged in the study of anatomy, and consequently much exposed to the noxious influence of cadaveric effluvia, and having also witnessed serious disease from septic inoculation, I have deemed it appropriate to mention a few peculiarities of this affection. In this, as in other things, a timely hint may protect the unwary from danger, and, at the same time, allay the needless alarm of the timid.

It has been often remarked that those who assiduously prosecute anatomical dissections, passing each day several hours in the midst of an unwholesome atmosphere, undergo some depreciation of healthy vigor. The putrid exhalations from the bodies taken into the system with every breath, occasionally cause a sallow hue of countenance, and generate dyspepsias and diarrhœas. In point of fact, with many persons, a flux from the bowels seems to be an initiatory step, an acclimation, as it were, to research in practical anatomy. This opinion is almost confirmed by the fact equally noticeable, that a temporary withdrawal from the cause of sickness gives relief, and in the majority of instances, no recurrence of the disease will take place even during a long continuance in dissections. Putrid effluvia, when concentrated and inhaled, give rise to constitutional disturbance; and though the preservative tendency of habit may render some immunity from their usual influence, adynamic fevers and dysenteries have been generated, and when decomposing materials, from whatever source derived, are inserted into the wounds, the local mischief and the contamination of the circulatiug fluids give origin to serious disease. The cook that caters for the taste of the epicure receives from high game painful eruptions and abscesses; and in thus finding the system infected by applications of a deleterious nature to the exterior of the body, we are surprised that lovers of tainted food should so frequently escape sickness; but such is the protective influence of habit in reconciling the palate to the dainty morsel, that the luxurious liver grows obese and gouty on food that would be insupportable and destructive to those unaccustomed to eat it in this state. Nor is a knowledge of this property of dead matter confined alone to the learned, for the vengeful savage, in deadly hate, hurls against his enemy the dart steeped in the fetid body of his murdered companion. Disastrous consequences have originated from fetid odors, when allowed to escape, either by accident or design, from bodies far advanced in decomposition; so that from the upheavings of cemeteries sudden and fatal diseases have had their birth.

But it is not the anatomist alone who suffers from putrescent matters; for it appears that in certain persons a peculiar aptitude to this disease exists. This predisposition, however, does not present itself at all times in the same individual; for, by some change of constitution, a person may be susceptible to its slightest influence, whilst on another occasion he will resist evident exposures. Concerning this topic, Dr. Gross, in his recent valuable treatise on surgery, hold this language: "There is no question that some persons are peculiarly prone to suffer from this poison. I am acquainted with a physician who was formerly much engaged in pathological researches, who rarely opened a dead body without having a dissecting boil on his hand, thumb or finger. Occasionally the consequences were more serious, the disease extending up the arm, along the course of the absorbents, as high as the axilla." Furthermore, many persons worn down by the arduous duties of student life, suffer from any slight abrasion. Direct absorption in these instances may not take place, but the local irritation generated by the constant maceration of the hands in putrid matter, may give rise to constitutional fever. This irritation may be thrown along the absorbents, even to the axilla; yet in such cases we never witness that sudden depression of vital resistance which is so characteristic of the virus into the general circulation. In the one case, the local mischief precedes the general disturbance, whilst in the other, the constitutional irritation over proportions the local inflammation; and, in the language of Mr. Travers: "The local inflammatory action is an unessential and subordinate feature of the disease in its severest form, the disease itself consisting of a direct prostration of the vital forces, marked by a preternatural excitement and rapid exhaustion." Besides, mere local abscesses at the point of contact of the matter, we occasionally find that painful tubercles may arise. Several eminent anatomists of our country have been afflicted with this rare form of the disease.

In discussing this question of animal poison, it becomes a subject of some moment to consider the circumstances under which this disease may be produced. The assertion has been made by high authority, that the effects of morbid animal matters could only be generated and made efficient at an early period after the vital powers have become extinct. Could such a theory be established, could it be clearly proved that this deadly poison, brought into activity ere vitality was dissipated, is soon to be evolved by putrefactive changes, the practical anatomist would have achieved a great boon. Mr. Travers, in his work on "Constitutional Irritation," confirming the view that the disease arises from the absorption of the fluids of fresh bodies, holds the following language: "Of all the examples of the disease which I have given or selected, it is remarkable that the subjects were recent. Not one had been buried, some were yet warm. Even of those in which, not inspection, but demonstration, was the object, the bodies were in a perfectly fresh state. If, as is probable, at the moment of expiring vital influence, or of deanimalization, new combinations give birth to a specific matter of contagion, it is to be presumed that the ultimate state of dissolution and decay which we term putrefaction, so alters its quality as to neutralize and render it inert. And it is in conformity with observation, that actual putrescency is in some degree a security from the effects of this species of injury." In the paragraph immediately succeeding that above quoted, he attempts to deduce an analogy from comparative pathology. Vegetables, at the expiration of life, are known to undergo such chemical changes that new combinations are generated, which must also disappear previous to the final decay annihilating the original structure; and the author concludes with the reflection that "chemical observation and analysis directed to the subject, would, and probably one day will throw light upon the fact that the first changes which take place in the animal body, after the utter extinction of the vital influence, produce a fluid more deleterious in its effects upon the living body than any which succeed it, and will also determine whether the fluids generated by disease, when undergoing these changes, possess more or less of this property."

Though the above opinion in regard to this malady obtains with many, and would be highly satisfactory in the use of prophylactic measures, we yet have reason to believe that fatal effects may result from subjects far advanced in decomposition, and even from the living. Innumerable accidents of this character prove that morbid poison may be induced from specimens long kept; and, in corroboration of the belief that morbid fluids from the living may produce disastrous consequences, I will offer the following quotation from a letter addressed to me by Dr. S. Roby, the able Professor of Anatomy in the University of Maryland. After making allusion to the general nature of this infection, he remarks: "My brother-in-law, Dr. Sharpe, of Boston, was also very ill for months. His case arose from poison received from living, not a dead subject. The main points are as follows: A dispensary patient, recently confined, came under his care. He saw her for the first time on Saturday, and was obliged to empty her bladder with the catheter. She evidently had puerperal peritonitis. On Sunday he used the catheter again. On Monday, while riding, he was suddenly attacked with intense headache, pain in the back, and severe rigor. I saw him on his return home, still in the chill, and complaining of great pain in the back. He supposed that he was about to have typhus fever, or small-pox. There being great nausea, I advised an emetic of ipecachuana, which he took. The pain, however, continued, and he was very ill through the night. The next morning, I asked him, 'if he had any patients with erysipelas?' He then told of the case he was attending. I examined his hands, and found on the end of his left forefinger an almost imperceptible abrasion. The case was clear to me. He was suffering from *morbid poison*. Soon the usual symptoms followed—intense restlessness, headache, pain in the back, delirium and prostration. In truth, his condition was an exact transcript of Morla's. A large abscess formed under his pectoral muscles, and he barely escaped with his life. Morla's case you saw. I had no doubt of its character from the beginning, and if you remember, predicted the course and event."

The case above alluded to as resembling Dr. Sharpe's, was that of a medical student, occurring in the Baltimore Infirmary, in the summer of 1857, during my term of service as house physician to that institution. On a sultry summer evening of that year, a poor sailor died of a cancerous affection of the stomach. He had lingered long, and an autopsy was held about 10 o'clock on the night succeeding his death. The body still retained warmth, and during the examination Mr. Morla received several abrasions on the left forearm, from the cut ends of the ribs. His hands were smeared with the secretions of the body, and at this time the contact of the matter gave no sense of irritation. At noon of the following day, he complained of chilly sensations, the conjunctivæ of his eyes were injected with blood, and his restlessness and jactitation evinced irritation of the nervous system. Not being satisfied with the unfavorable symptoms in this case, I sent a messenger for Dr. Miltenberger, the visiting surgeon of the Infirmary, who, arriving late in the afternoon, found Mr. Morla in an active delirium. He had great heat of skin, and a bounding pulse. At this time, no signs of inflammation were evident about the abrasions. As his cerebral symptoms were untoward, and his pulse justified the procedure, he was bled from the arm. After losing several ounces of blood, he fell asleep, and on the succeeding morning was quite rational.

At this period of the disease, we could approach a correct diagnosis; for he now complained of irritability and numbness of the left arm, attended with excessive pain in the axilla and beneath the pectoral muscles. Recurring to the autopsy of the previous night, conjoined with the extreme distress and prostration, we were obviously dealing with poison from animal matter.

Mr. Morla suffered much, and, though carefully attended, many weeks elapsed before recovery took place. His frame, naturally robust, had to contend, not only with the sedative influence of the poison, but large abscesses having speedily formed under the pectoral muscles and down the arm as far as the elbow, the continued suppuration from such an extensive surface almost drained the fountain of life.

Since coming to New Orleans, I have witnessed an instance of septic inoculation similar to the one above detailed. A man suffering from the effects of intemperance, inflicted a mortal wound on his wife, and then attempted suicide. Both man and wife came to the Charity Hospital in a mutilated condition, and the latter, lingering a few days, died. Mr. Shelby, a resident student of the Hospital, having charge of the ward to which she was consigned, assisted at the coroner's inquest. From an abrasion on the right hand, the toxic material was absorbed.—The usual phenomena—chill, pain in the axillæ, and fever—soon supervened; and abscesses discharging profusely, formed about the axillary glands, and many weeks elapsed before recovery took place.

In both instances just mentioned, the subjects from which the malady resulted were recently dead; and, had we not opposing evidence, I would subscribe to the testimony of some, that the peculiar poison "produced by the textures before their vital properties and cohesions are quite extinct," could be only rendered baneful at an early period after death.

The symptoms attending this malady are generally characterized by extreme depression of the nervous system. At the point of contact, there is rarely any evidence of inflammation beyond slight redness; and so agonizing and violent are the effects, that the local action can only be regarded as a secondary and unimportant feature. Indeed, the severity of the rigors, the intensity of the pain, the nausea and delirium, all testify that a a blood poison has been absorbed. In Mr. Morla's case, little irritation was shown about the abrasion till the more serious trouble in the axilla had declared itself. On the third or fourth day after the reception of the poison, a small vesicle appeaerd on the wrist of the left arm, which eventually desquamated without attracting further attention.

It has been supposed that the disease was allied to the malady of which the infected body died. But such a view is erroneous, since it is well ascertained that similar results have ensued from the examination of bodies dead by accident; yet it must be admitted that diseases attacking serous surfaces have manifested the greatest disposition to cause infection. In truth, in all bodies recently dead, this poison exists in various grades and activity, irrespective of any form of disease. And furthermore, the symptoms of this disease somewhat resembling those of pestilential epidemics, can only be understood by the supposition that the circulating fluids are contaminated, and that the nervous system is oppressed by a peculiar irritant. In some cases, this virus must be generated long before vitality is extinct; for it is thus only that we can harmonize the above views with the fact that the living occasionally cause inoculation. And in those isolated cases where poison has been absorbed from handling pathological specimens, we must conclude that the preservative fluid, whether alcohol or other menstrua, in arresting putrefactive alterations, retained the septic element, to be rendered effective and deadly when brought in contact with the abrased hand of the curious.

In the majority of the cases on record, the poison was taken from bodies recently dead, and its virulence manifesting itself within eighteen hours after reception, the sad story of their issue is told in speedy death or a convalescence.

It is well worthy of remark, that the axillary glands are the foci around which inflammation originates destructive action on the tissues. It appears that nature offers in this situation some resistance to the offending material; but, so diffusive and blighting is this subtle agent, so rapidly does it pervade the organism, that the most robust frame is made to totter under its toxic effect.

In a disease always formidable, and often extremely fatal, it behooves us to understand the preventive measures placed in our power. If, as some suppose, the virulence of the poison is proportionate to the freshness of the body dissected, we who study anatomy in New Orleans should maintain the greatest caution; for none of the material used in our demonstrations has ever been buried. But, happily, though the heat of climate and the constitutions of our students, may predispose to disease, I have never known an instance of poison to occur in the dissecting rooms of the University of Louisiana.

In consequence of the high degree of temperature peculiar to this latitude, great care is maintained in the ventilation of our rooms, and in the speedy withdrawal of all offensive matter.—Moreover, as every dead body brought into our dissecting rooms has been preserved with a solution of the chloride of zinc, the student need not apprehend any danger from poison, for no other disinfectant creates such alterations in the tissues of the body; and thus far, the records of medicine furnish no instance of inoculation following the dissection of bodies injected with this valuable agent. In this respect, New Orleans presents to the student of medicine unsurpassed facilities for the acquisition of knowledge in anatomy. Possessed of ample material, which can be reclaimed from the destructive tendency of disease and climate, not only during our short and mild winters, but even during the heat of midsummer, when epidemics are rife, our dissecting rooms can satisfy the avidity of every earnest worker in this department of science. Although persuaded that inoculation can scarcely take place, we recommend to students, in order to allay fear, in case of cuts and abrasions on their hands, to resort immediately to the use of water and sucking the wounded part with the lips. We eschew the use of caustics and other irritants; for, from their free application to simple injuries, troublesome sores have been produced. Besides, as the action of this poison is almost coincident with its insertion into the wound, it would be unavailing.

When the specific virus has been absorbed, the disease resembles other affections of this class; and in our efforts to bring about a favorable issue, we should have recourse to such means as may sustain the flagging powers of life. The patient, in point of fact, has his own cure in his capacity of vital resistance. The strong man will quickly divest himself of the deadly poison, whilst the feeble will sink under the depressing cause. Every animal poison, whether originating from venomous reptiles and insects, or from the dead body, engenders a depressing influence on the vital powers; and whatever tends to facilitate the introduction of the virus into the circulation, will add to the dangers of the disease. In all cases of blood poison, the struggle that ensues between the action of the poison and vital resistance so alarms the functions of life, that many of the symptoms of inflammation are simulated. Few persons can undergo depletory measures, which would diminish the vigor of the constitution, and increase the rapidity of absorption; for this disease, like erysipelas, typhus, and similar affections, compels us to do nothing that may weaken the inherent strength of the patient. Rarely would we think of resorting to the lancet in erysipelas; for though its outburst with fever, pain, delirium, and a full pulse, may tempt us to such a procedure, yet in the majority of instances, a short lapse of time will evince the fact, that the calm is approaching when judicious care and every ounce of blood are requisite to carry the patient to a favorable termination. As yet, we are ignorant of any constitutional remedies that can be of positive efficacy when directed against septic poisons. In one of the cases referred to in this paper, that of Mr. Morla, the iodide of potassium was administered, but this agent exerted no antidotal properties. Perhaps further investigation may elicit knowledge corroborative of the information already set before the medical world in reference to the depurative effects of bromine and iodine in cases of animal poison. In the incipiency of this disease, palliative treatment, and the use of such measures as will alleviate pain and prevent suffering, should be the sole aim of the judicious surgeon. Proper vigilance will indicate the time for supporting the patient amid the adverse chances of the disease, which, like kindred ailments, much resembles in its course an oceanic voyage that, barring accidents, will have a happy termination.—*N. O. Med. & Surg. Journal.*

